# *In vivo* nanoparticle-based T cell imaging can predict therapy response towards adoptive T cell therapy in experimental glioma

**DOI:** 10.7150/thno.87248

**Published:** 2023-09-25

**Authors:** Jessica Hunger, Katharina Schregel, Berin Boztepe, Dennis Alexander Agardy, Verena Turco, Kianush Karimian-Jazi, Ina Weidenfeld, Yannik Streibel, Manuel Fischer, Volker Sturm, Rachel Santarella-Mellwig, Michael Kilian, Kristine Jähne, Katharina Sahm, Wolfgang Wick, Lukas Bunse, Sabine Heiland, Theresa Bunse, Martin Bendszus, Michael Platten, Michael O. Breckwoldt

**Affiliations:** 1 Neuroradiology Department, University Hospital Heidelberg, Heidelberg, Germany.; 2 Clinical Cooperation Unit Neuroimmunology and Brain Tumor Immunology, German Cancer Consortium (DKTK) within the German Cancer Research Center (DKFZ), Heidelberg, Germany.; 3 Department of Neurology, Medical Faculty Mannheim, Mannheim Center for Translational Neurosciences, Heidelberg University, Mannheim, Germany; 4 European Molecular Biology Laboratory (EMBL), Heidelberg, Germany.; 5 Clinical Cooperation Unit Neurooncology, DKTK within DKFZ, Heidelberg, Germany.; 6 Department of Neurology, National Center for Tumor Diseases (NCT), Heidelberg University Hospital, Heidelberg, Germany.; 7 DKFZ-Hector Cancer Institute at University Medical Center Mannheim, Mannheim, Germany.

**Keywords:** glioma, tumor microenvironment, iron oxide nanoparticles, immunotherapy, adoptive T cell therapy, non-invasive treatment monitoring

## Abstract

**Rationale:** Intrinsic brain tumors, such as gliomas are largely resistant to immunotherapies including immune checkpoint blockade. Adoptive cell therapies (ACT) including chimeric antigen receptor (CAR) or T cell receptor (TCR)-transgenic T cell therapy targeting glioma-associated antigens are an emerging field in glioma immunotherapy. However, imaging techniques for non-invasive monitoring of adoptively transferred T cells homing to the glioma microenvironment are currently lacking.

**Methods:** Ultrasmall iron oxide nanoparticles (NP) can be visualized non-invasively by magnetic resonance imaging (MRI) and dedicated MRI sequences such as *T*_2_* mapping. Here, we develop a protocol for efficient *ex vivo* labeling of murine and human TCR-transgenic and CAR T cells with iron oxide NPs. We assess labeling efficiency and T cell functionality by flow cytometry and transmission electron microscopy (TEM). NP labeled T cells are visualized by MRI at 9.4 T *in vivo* after adoptive T cell transfer and correlated with 3D models of cleared brains obtained by light sheet microscopy (LSM).

**Results:** NP are incorporated into T cells in subcellular cytoplasmic vesicles with high labeling efficiency without interfering with T cell viability, proliferation and effector function as assessed by cytokine secretion and antigen-specific killing assays *in vitro*. We further demonstrate that adoptively transferred T cells can be longitudinally monitored intratumorally by high field MRI at 9.4 Tesla in a murine glioma model with high sensitivity. We find that T cell influx and homogenous spatial distribution of T cells within the TME as assessed by *T*_2_* imaging predicts tumor response to ACT whereas incomplete T cell coverage results in treatment resistance.

**Conclusion:** This study showcases a rational for monitoring adoptive T cell therapies non-invasively by iron oxide NP in gliomas to track intratumoral T cell influx and ultimately predict treatment outcome.

## Introduction

Glioblastoma (GBM), the most common and aggressive primary brain tumor in adults, has a dismal prognosis with a poor median survival of ~15 months after diagnosis 1,2. GBM is characterized by a highly immunosuppressive tumor microenvironment (TME) with high myeloid content. These myeloid cells suppress T cell effector function and lead to T cell exhaustion and anergy 2. In the past decade several new immunotherapies based on adoptive T cell transfer (ACT) have been introduced into clinical practice 3, 4. This includes T cell receptor (TCR)-transgenic T cells and chimeric antigen receptor (CAR) T cells that can bind to unprocessed antigens on the cell surface and do not require additional co-stimulatory signals. CD19 CAR T cells are now the standard of care for certain types of refractory lymphomas and leukemias 5 and solid cancer indications for CARs have shown promising results 6. There are also multiple preclinical studies and ongoing clinical trials to support that CARs can be effective in glioma 7-9, however, CAR T cells have not entered routine clinical practice for GBM 10-13. Sufficient T cell recruitment to the tumor and intratumoral distribution are likely major determinants of treatment outcome during ACT, but currently methods to monitor ACT non-invasively are lacking. Clinical monitoring is mainly restricted to invasive tissue sampling or structural imaging information. Preclinical studies have shown heterogeneous responses to immunotherapy in glioma 14. In addition, pseudoprogression (PsPD), an apparent enlargement of the tumor mass due to immune cell infiltration, cannot be easily differentiated from tumor growth by conventional magnetic resonance imaging (MRI) 15. This creates the need for the development of non-invasive immune cell tracking approaches to monitor immunotherapy and adapt therapy accordingly. MRI is the most important clinical diagnostic tool to monitor brain tumors. Ultrasmall superparamagnetic iron oxide (USPIO) nanoparticles (NP) can serve as MRI contrast agents and can be quantified by *T*_2_* relaxometry. It is also well established that USPIOs allow tracking of various cell types including macrophages, dendritic cells, tumor cells or stem cells 16-21. These cell types have high phagocytic activity and can be labeled *in vitro* or systemically after intravenous injection of USPIOs 22. T cells as non-phagocytic cells however, are not readily transduced by USPIOs, so dedicated labeling or transfection protocols are required 23. In this study, we establish a protocol using a dextran coated iron oxide NP to track adoptively transferred, tumor antigen-specific transgenic or CAR-T cells. We show that T cell distribution within established intracranial gliomas is associated with treatment response and thus allowed response prediction of adoptive cell therapy.

## Results

### Establishing a T cell labeling protocol using iron oxide nanoparticles

**Figure 1:**
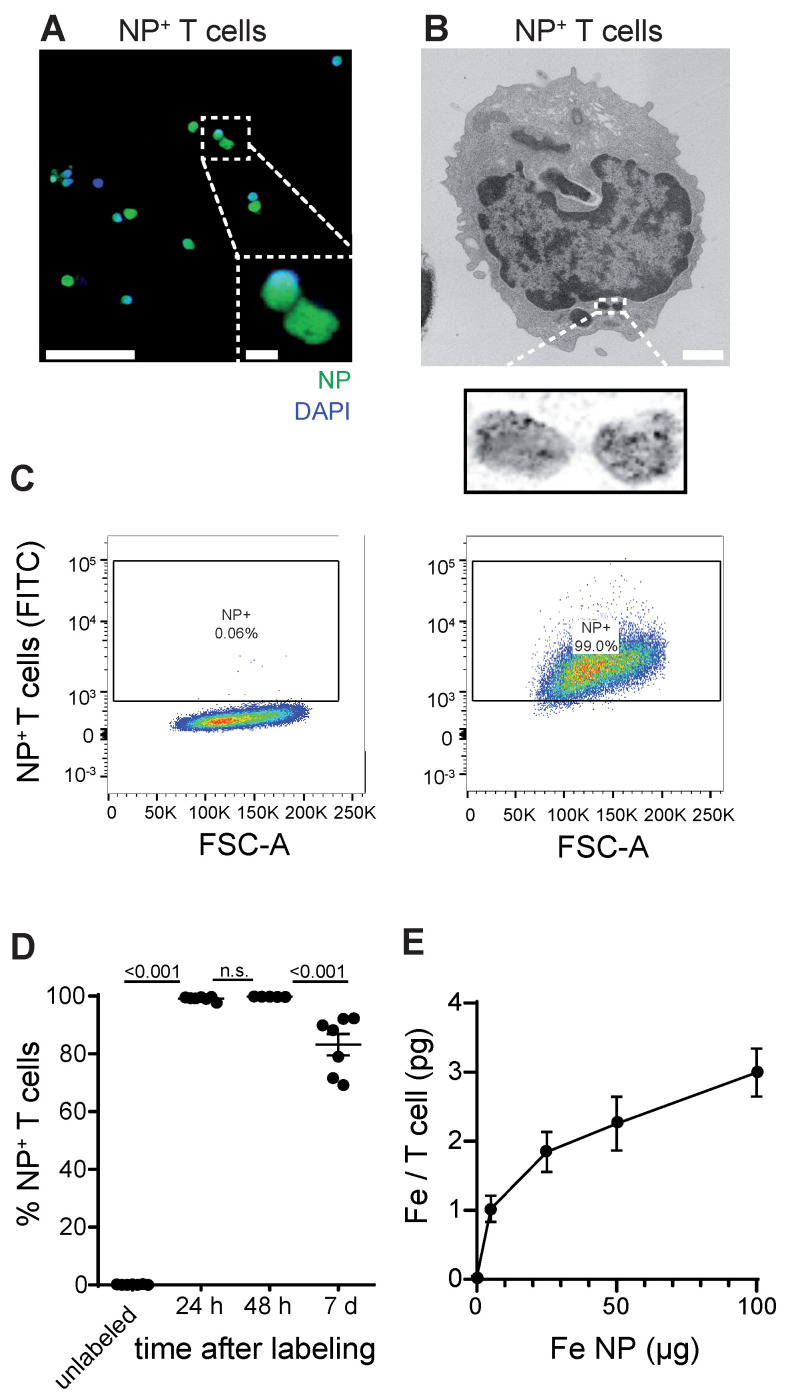
** A:** Confocal image of iron oxide NP labeled T cells **B:** Transmission electron microscopy (TEM) of NP labeled primary murine T cells. **C:** Flow cytometry plots of unlabeled and iron oxide NP labeled primary murine T cells after 24 h incubation with NP *in vitro* **D:** Quantification of iron oxide NP labeling efficacy after 24 h, 48 h and 7 days, assessed by flow cytometry **E:** Quantification of iron uptake per T cell after 24 h incubation with iron oxide NP *in vitro*, assessed by ICP-OES. Scale bars are 20 µm in confocal microscopy image, 2 µm in inset and 500 nm in EM.

First, we established a protocol to label primary murine CD8^+^ T cells with a dextran coated ultrasmall iron oxide NP (Molday ION^TM^ EverGreen, 35 nm diameter) by co-incubation of T cells with NP under modest stimulatory conditions with Interleukin 2 (IL-2), CD3 and CD28. To dissect NP localization within T cells we performed confocal microscopy that showed robust intracellular NP uptake of primary murine T cells (**Figure 1A, Figure S1A**). Subcellular localization was further assessed by transmission electron microscopy (TEM), which showed localization of NP inside cytoplasmatic vesicles (**Figure 1B, Figure S1B**). We achieved high labeling efficiency of 99.1% ± 0.3% *in vitro* after 24 h as assessed by flow cytometry (n = 7 T cell cultures, **Figure 1C-D**). 7 days after T cell loading, labeling efficiency remained high at 83.2% ± 3.7%, which demonstrates that long-term labeling of T cells with this iron oxide NP is possible (**Figure 1D**). Inductively coupled plasma atomic optic emission spectrometry (ICP-OES) revealed that each T cell takes up ~2.3 ± 0.2 pg Fe when exposed to 50 µg Fe NP following a saturation kinetic (**Figure 1E**). To test the imaging properties of our iron oxide NP, various NP concentrations were measured by 9.4 T MRI. *T*_2_* relaxation times were assessed based on a multi gradient echo (MGE) sequence and showed an exponential drop of *T*_2_* relaxation time with increasing iron NP concentrations (**Figure S1C,D**).

### Iron Oxide NP labeling does not affect T cell viability or functionality

**Figure 2:**
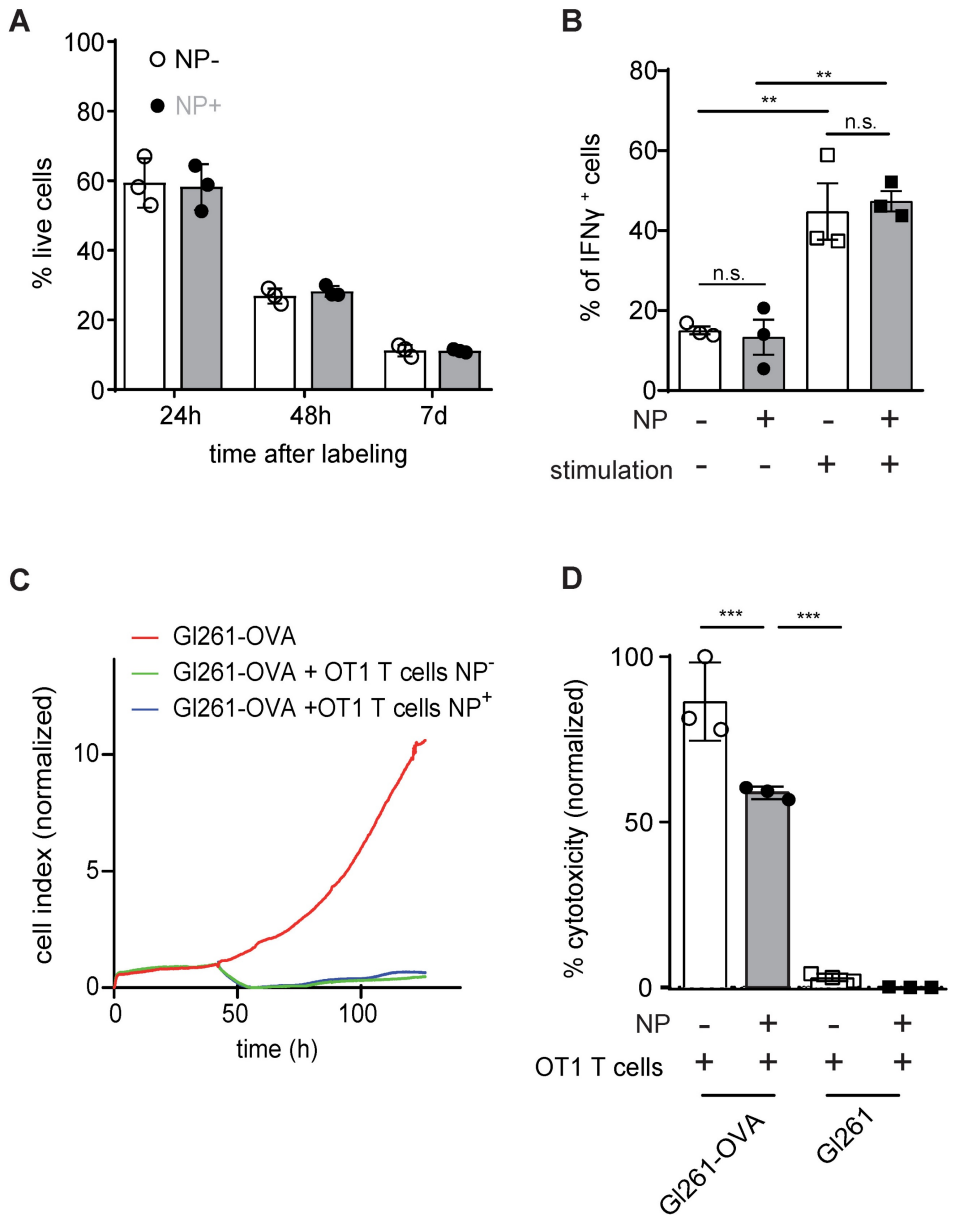
** A**: Quantification of T cell viability comparing unlabeled primary murine T cells and iron oxide NP labeled T cells after 24 h, 48 h and 7 days of labeling *in vitro*, as assessed by flow cytometry **B:** IFN γ secretion of unlabeled *vs* iron oxide NP labeled T cells after 24 h of labeling, assessed by flow cytometry **C:** Antigen-specific killing activity of unlabeled *vs* iron oxide NP labeled murine OT1 T cells co-incubated with Gl261-Ova target cells, assessed by RTCA. The experiment shows the mean of n = 3 independent biological replicates **D**: LDH release assay measuring antigen-specific killing activity of unlabeled *vs* iron oxide NP labeled OT-1 T cells co-cultured with Gl261-Ova cells or Gl261 cells

Next, we assessed putative toxicity of our labeling approach on T cells. Importantly, NP labeling of primary murine T cells with 50 µg of iron NP did not impede T cell viability after 24 h, 48 h or 7 days after NP labeling (**Figure 2A**). IFNγ cytokine production as a measure of T cell functionality was also unaltered (**Figure 2B**). Antigen-dependent killing activity after NP labeling of murine primary T cells was assessed by real time cell analysis (RTCA) and the lactate dehydrogenase (LDH) release assay (**Figure 2C,D**). Both assays confirmed that NP labeled T cells are functionally unaffected and efficiently kill antigen presenting tumor cells *in vitro* in an epitope specific manner (**Figure 2C,D**).

### Extending the labeling protocol to CAR T cells

To further validate our labeling protocol and assess its translational potential we extended our NP labeling approach to CAR T cells. Here, we found that human CD19 CAR expressing T cells can also be efficiently labeled by our protocol (**Figure S2A-C**).

We further assessed human CD19 CAR T cell killing activity by co-incubation with CD19 expressing NALM6 cells which showed that NP labeled CD19 CAR T efficiently killed ~89% ± 2.9% of the target cells compared to 98.9% ± 0.02% by unlabeled CAR T cells (p > 0.05, **Figure 2C**).

### Nanoparticle labeled T cells can be visualized intracranially

**Figure 3:**
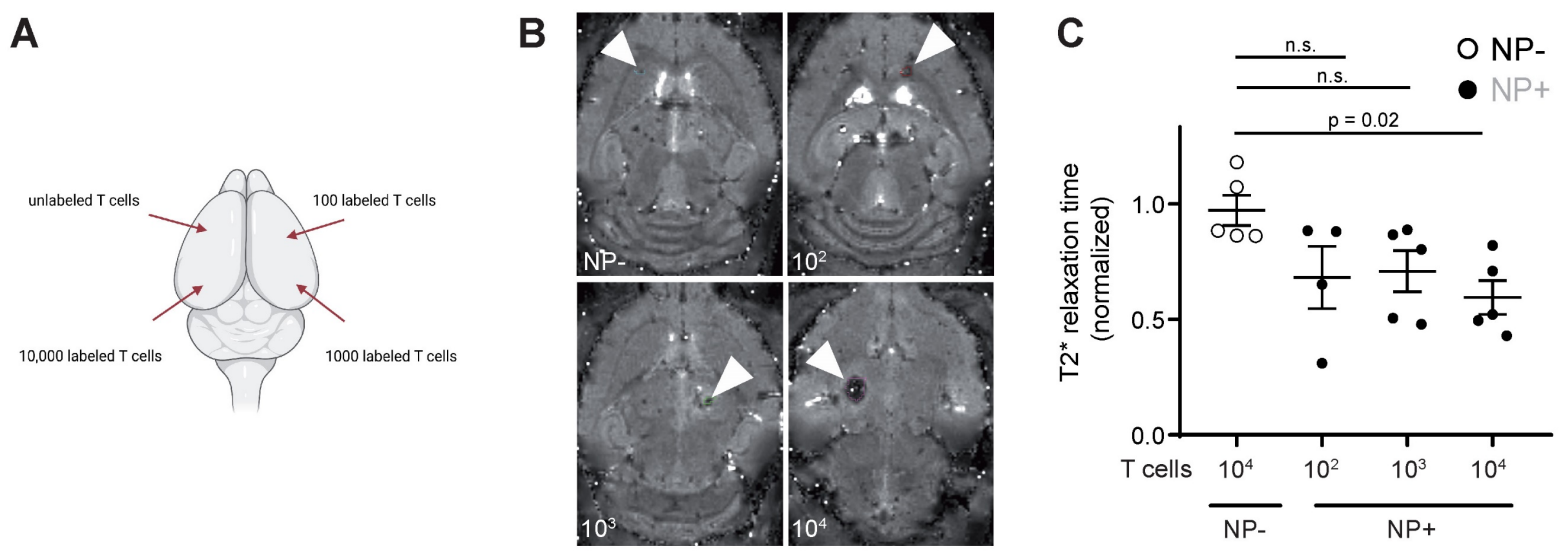
** A:** Schematic visualization of the four intracranial T cell injection sites. Injections were performed with 10.000 unlabeled or NP^+^ T cells (100, 1.000 or 10.000 T cells) **B:** *T*_2_* images of intracranial T cell injections. Arrowheads indicate the injection site** C:** Quantification of *T*_2_* time of intracranial, iron oxide NP labeled T cell injections. Relaxation time was normalized to the adjacent healthy brain tissue

To assess the detection sensitivity of NP labeled T cells by MRI, different NP labeled T cell concentrations (unlabeled *vs* 10^2^ - 10^4^ NP^+^ T cells) were injected into the parenchyma of mouse brains *ex vivo* to identify the detection limit of labeled T cells (**Figure 3A**). 10^4^ NP labeled T cells could be visually detected by *T*_2_* imaging and showed a significant shortening in *T*_2_* relaxation time compared to unlabeled T cells (**Figure 3B**). Therefore, the detection limit of NP labeled T cells by MRI in our approach is ~10.000 T cells.

### Tracking T cell dynamics in murine glioma following ACT

**Figure 4:**
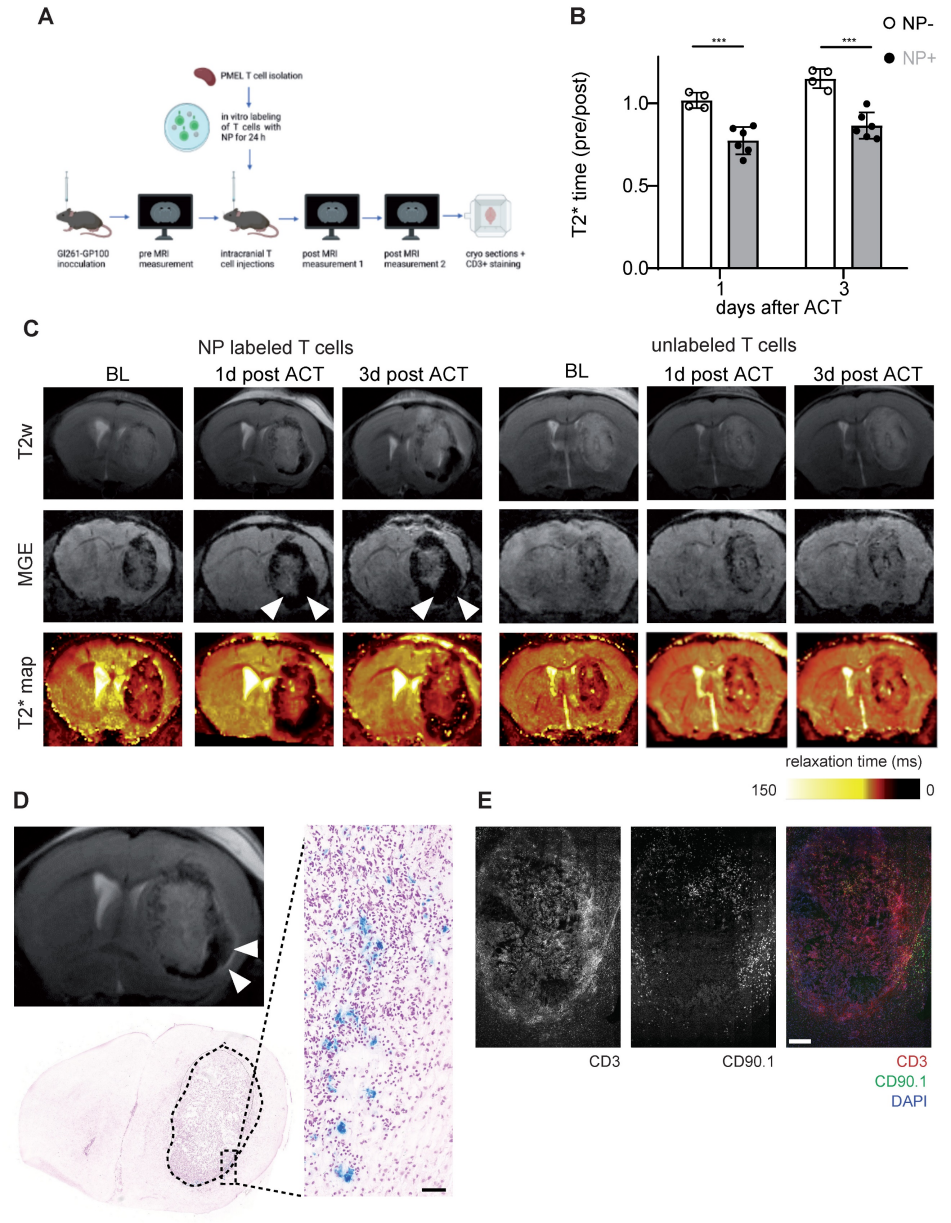
** A:** Schematic experimental outline of intratumoral T cell injections and subsequent MRI measurements **B:** Quantification of tumor relaxation time 1 and 3 days after intratumoral ACT with iron oxide NP labeled T cells or unlabeled T cells from *T*_2_* maps. Relaxation time was normalized to the baseline (pre) measurement before ACT **C:** T2w, MGE and *T*_2_* map of mouse brain with intratumoral ACT (iron oxide NP labeled *vs* unlabeled T cells) **D:** Prussian blue staining after intratumoral ACT with iron oxide NP labeled T cells in comparison to MR image **E**: Immunohistochemistry of CD3^+^ / CD90.1^+^ / DAPI co-staining, imaged by confocal microscopy.

After demonstrating that NP labeled T cells can be visualized non-invasively by MRI in the healthy mouse brain, we performed T cell tracking in the murine Gl261 glioma model. 13 days after Gl261-gp100 tumor implantation mice were imaged by MRI to quantify the tumor *T*_2_* relaxation time at baseline (BL). Antigen-specific PMEL T cells were isolated from healthy donor mice, labeled with iron oxide NP *ex vivo* and used for adoptive T cell transfer (ACT). First, we assessed whether T cells could be monitored following intravenous transfer. However, T cell recruitment to the tumor was insufficient and most T cells homed to the spleen and lymphnode (**Figure S3A**). Next we probed intraventricular adoptive transfer (**Figure S3B,C**). However, in contrast to previous reports 24, intraventricular T cell administration in our model did not lead to sufficient T cell recruitment into the intraparenchymal glioma nor therapeutic efficacy (**Figure S3C,D**). In a third approach Gl261 glioma bearing mice were treated with a single intratumoral injection of CD90.1^+^ T cells and monitored longitudinally one day and three days after ACT (**Figure 4A,B**). T2* relaxation times of the tumor remained unaltered after ACT in animals receiving unlabeled T cells whereas there was a significant *T*_2_* relaxation time drop in mice that received NP-labeled T cells (**Figure 4B,C**). This indicates that shortened relaxation times are not caused by the intratumoral injection *per se* or induced microbleedings but rather specific to the administered NP labeled T cells. These differences between NP labeled T cells and unlabeled T cells persisted over at least 3 days post ACT (**Figure 4C**). To further validate MRI results, we performed correlated histology and immunohistochemistry (IHC). Brains were cryosectioned and stained for Prussian blue and CD3^+^ / CD90.1^+^ co-staining to visualize endogenous and adoptively transferred T cells (**Figure 4D,E**). In line with the MRI results, iron stained by Prussian blue was mainly present in the tumor-stroma border zone (**Figure 4D**). Moreover, IHC of consecutive sections confirmed CD3^+^ T cell accumulations in the Prussian blue positive areas. Also, most of these T cells were positive for CD90.1, indicating that these T cells represent the adoptively transferred T cell population. Additional flow cytometric analysis of transferred T cells confirmed that CD90.1^+^ T cells were phenotypically active and showed unaltered exhaustion markers for PD-1 and Tim3*,* whereas only Lag-3 showed a modest downregulation in NP labeled T cells (**Figure S3E**). To validate our data in a second model, the subsequent experiments were performed using the more potent Ovalbumin (Ova) model antigen.

### Tumor response can be predicted based on early intratumoral T cell distribution

**Figure 5:**
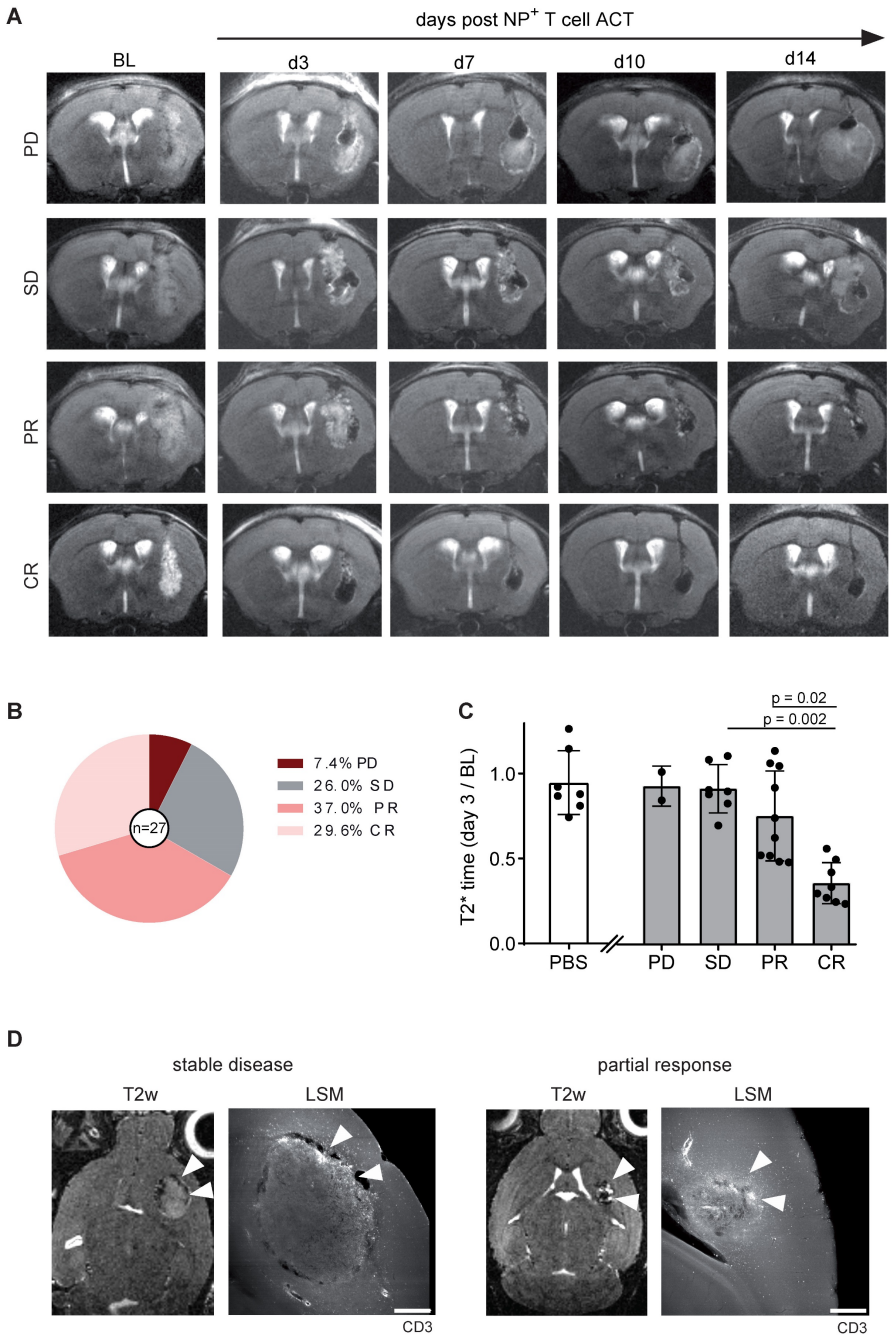
** A:** T2w MR images OVA-Gl261 bearing mice following intratumoral iron oxide NP labeled OT-1 ACT. Representative examples of progressive disease (PD), stable disease (SD), partial response (PR) and complete response (CR) are shown. **B:** Response towards ACT with OT-1 T cells. n = 27 mice. Pooled data from two independent biological experiments **C:** T2* relaxation times of the tumor within the response groups. PBS was injected in a negative control group. **D**: T2w MRI of intratumorally injected, NP labeled OT-1 T cells in Gl261-Ova model on day 14 after ACT in correlation to iDISCO cleared LSM images of CD3^+^ T cells. Arrowheads indicate sites of T cells accumulations. Scale bar is 500 µm.

Gl261-Ova tumor bearing mice were injected with 1 x 10^6^ iron oxide NP labeled OT1 T cells intratumorally 14 days after glioma engraftment (BL). Tumor growth and *T*_2_* relaxation times were assessed over a time period of 14 days after ACT (**Figure 5A**). Tumor response was classified according to RANO using clinical response criteria (progressive disease (PD), stable disease (SD), partial response (PR) and complete response (CR)) 14. Interestingly, mean T2* tumor relaxation time on day three after ACT correlated with tumor response: Mice with a decreased relaxation time of the tumor showed complete or partial response on day 14 after ACT, whereas an unaltered tumoral *T*_2_* relaxation time compared to BL was associated with treatment resistance (PD or SD; **Figure 5B,C**). To further assess the T cell distribution in the TME, brains were further processed for whole brain clearing and imaged using light sheet microscopy (LSM). Interestingly, tumor response was associated with T cell coverage of the entire tumor area, whereas T cells were restricted to the tumor border in treatment resistant mice (**Figure 5D**). These results demonstrate that T cell imaging of intratumoral T cell distribution is linked to response and resistance at an early time point after ACT and thus can be employed for ACT response monitoring.

## Discussion

Glioblastoma is a highly malignant brain tumor with heterogeneous response to immunotherapy in preclinical studies and clinical immunotherapy trials 4, 25. Currently, there is no clinical modality to monitor immune cell recruitment to the TME. However, imaging intratumoral immune cell distribution non-invasively could facilitate immunotherapy monitoring for solid tumors including glioma. In this study we establish a robust protocol for labeling primary murine T cells with a diagnostic iron oxide NP and show that T cell labeling does not affect T cell viability or functionality as assessed by cytokine secretion (IFNγ secretion) and antigen-specific killing assays. Furthermore, we confirm that this approach is also applicable to human CD19 CAR T cells. This demonstrates that a NP based T cell tracking approach could be feasible in clinically relevant treatment paradigms. Previous work by Thu et al. established a labeling protocol for T cells using Ferumoxytol (FeOx) and heparin/protamine nanocomplexes 26. Furthermore, Kiru et al. recently developed a microfluidics based system for FeOx labeling of CAR T cells that used mechanical cell volume exchange and convective transfer to label T cells 19. In comparison, our labeling approach does not require chemical or mechanical manipulation and achieves high T cell labeling efficiency > 99% using modest T cell activation with IL-2, CD3 and CD28.

Our protocol could detect ~10.000 cells at 9.4 T and achieved ~2 pg Fe NP uptake per T cell. Therefore, our approach cannot claim single cell resolution, but likely detects clusters of T cells or T cell accumulations. This is in line with recent reports using ferucarbotran and magnetic particle imaging (MPI) which showed a detection limit of 50.000 cells with an uptake of 1 pg Fe / cell 27. In addition, Molday ION BioPAL has the advantage that it contains a fluorescent conjugate that allows confirmation of labeling efficiency by flow cytometry or confocal microscopy *in vitro* 17. By correlating tumor response over time with the tumor *T*_2_* relaxation time, we demonstrate that the prediction of response or resistance is possible already at an early time point after ACT. Tumors that showed PR or CR exhibited a strongly reduced *T*_2_* relaxation time of the tumor, which suggests that a uniform T cell distribution within gliomas is necessary for efficient tumor cell clearing, whereas resistant tumors showed T cell compartmentalization at the tumor border. The exact mechanism of local T cell sequestration remains to be investigated, but both active tumor immune escape, mechanical redistribution or active TME suppression of transferred T cells seem plausible.

Our NP approach for T cell tracking could be further extended to additional immune cell populations: It has recently been shown that combining iron oxide-based NP tracking with gadolinium (Gd) based cell tracking in a dual colour tracking approach is possible 28. Hence, tracking myeloid cells in conjunction with CD4^+^ or CD8^+^ T cells at the same time or simultaneous T cell tracking of different subpopulations is likely possible.

NP labeling could furthermore be translated to other immune cell populations such as macrophages, which have been shown to exhibit NP uptake by macropinocytosis and phagocytosis *in vivo* 29 and macrophage imaging has been shown to be predictive of immunotherapeutic efficacy 30.

Our study has several limitations: In immuno-oncology, it is well established that immunotherapy outcome is not only determined by T cell infiltration and distribution, but also by T cell activity and exhaustion 31. While non-invasive, NP based T cell tracking provides information on spatial and temporal dynamics of T cells, it does not provide information on T cell phenotype and functional state including exhaustion, which is a limiting factor for non-invasive NP tracking. Information regarding T cell activation status could be gathered by MRI using fusogenic liposomes, which neutralize reactive oxygen species on activated T cells and produce paramagnetic 2,2,6,6-tetramethylpiperidine 1-oxyl (TEMPO) radicals, which can be visualized by MRI 32.

Moreover, our NP approach requires *ex vivo* labeling and therefore only detects adoptively transferred T cells, while omitting information on endogenous T cell dynamics. Also, the NP label is likely diluted during T cell proliferation. Our study does not reveal the specific NP uptake mechanism in T cells. TEM showed NP localization within cytoplasmatic vesicles, suggesting an endocytotic uptake pathway such as macropinocytosis 33. Macropinocytosis has been suggested as an uptake mechanism of NP in various immune cell types including T cells 34, 35 and is therefore a likely uptake mechanism of the iron oxide NP in T cells.

In addition to immune cell tracking based on MRI, there have recently been several additional approaches to visualize T cells based on positron emission tomography (PET) 36, 37. CD8^+^ minibodies have the advantage that endogenous T cells can be detected as well, however this tracking approach is not antigen-specific and requires the use of radiotracers. Anti-PD1 treatment has been monitored using the T-cell-specific PET agent [18F] F-AraG in order to visualize activated T cells in a murine colon adenocarcinoma model, however, this approach has not been translated to clinical practice 38.

In summary, our study establishes a novel approach for labeling of tumor epitope specific CD8^+^ or CAR T cells using iron oxide NP. We show that T cell labeling does not impact T cell viability or function and enables non-invasive, intratumoral visualization of adoptively transferred T cells over time, which can function as a predictor for response or resistance to cancer immunotherapy. We envision that our T cell tracking approach could also be used beyond monitoring immunotherapy in glioma and might be applicable to monitor T cell dynamics in other solid tumor entities as well as (neuro-)inflammatory conditions.

## Methods

### Mice

Specific and opportunistic pathogen free (SOPF) female C57Bl/6J mice were purchased from Janvier Laboratories at the age of 6-10 weeks. PMEL mice carry a rearranged T cell receptor transgene specific for the mouse homologue (pmel-17) of human SILV (gp100) and were used as donor mice for primary murine T cells in the gp100 model. OT-1 mice carry a transgenic T cell receptor that was designed to recognize ovalbumin residues 257-264 in the context of H2Kb and were also used as donor mice for T cells in the Ova model. Animals were checked daily for any neurological signs, weight loss or discomfort and terminated when termination criteria were reached. All animal protocols were performed in compliance with the laboratory animal research guidelines and were approved by the governmental authorities (animal protocols: G27-17 and G35-22, regional administrative authority, Regierungspräsidium Karlsruhe, Germany).

### Cell lines

Gl261 cells were purchased from the National Cancer Institute (NCI) and cultured in Dulbecco's modified Eagle's medium (DMEM) supplemented with 10% fetal bovine serum (FBS) and 100 U / mL penicillin and 100 µg / mL streptomycin (all Sigma-Aldrich) at 37 °C, 5% CO_2_. Gl261 cells were routinely tested for contamination by multiplex cell contamination test (Multiplexion GmbH). Cells were stored using a 9:1 mixture of DMSO and FBS in liquid nitrogen for long-term storage and thawed using pre-warmed cell culture medium. For passaging, cells were detached using trypsin-EDTA (Gibco), stopped by cell culture medium. Gl261 cells were not passaged more than ten times. The Gl261-OVA cell line was generated by transfecting Gl261 cells with pMXS-OVA-IRES-blasticidine as previously described 2. The Gl261-gp100 cell line was generated by cloning the open reading frame of murine gp100 to the pCCL.PPT.SFFV.MCS.IRES.eGFP.WPRE-vector backbone. To generate lentiviral particles HEK293T cells were co-transfected with the gp100 containing vector and the corresponding packaging plasmids (pMDLg / pRRE#54, pRSV-Rev and pMD2.VSVG). Released lentiviral particles were purified and concentrated by ultracentrifugation and used to transduce Gl261 cells in the presence of 8 mg / mL polybrene (Merck Millipore, Darmstadt, Germany). The medium was changed 24 h after transduction. During expansion, cells were repetitively sorted for high GFP expression. Prior to tumor injection, GFP and gp100 expression were confirmed by FACS and qRT-PCR, respectively.

### Tumor cell inoculation

Cells were detached from the cell culture flask using accutase (Sigma Aldrich), stopped by PBS. Mice were anesthetised by a mixture of 100 mg / kg Ketamin, 20 mg / kg Xylazin in sterile 0.9% NaCl and 1 x 10^5^ Gl261 tumor cells were diluted in 2 µL sterile PBS (Sigma-Aldrich) and stereotactically implanted into the right basal ganglia of 7-12 week old female C57Bl/6J mice (coordinates: 2 mm right lateral of the bregma and 1 mm anterior to the coronal suture with an injection depth of 3 mm below the dural surface) using a 10 µL Hamilton micro-syringe driven by a fine step stereotactic device (Stoelting).

### MR imaging

Multiparametric MR imaging was performed on a 9.4 Tesla horizontal bore small animal NMR scanner (BioSpec 94/ 20 USR, Bruker BioSpin GmbH, Ettlingen, Germany; gradient strength 675 mT / m) with an 8.4 cm volume coil for transmission and a four-channel phased-array surface receiver coil. MR imaging included a standard RARE T2-w sequence to monitor tumor volume (T2-w parameters: 2D sequence, 78 µm in-plane resolution, TE: 33 ms, TR: 2500 ms, flip angle: 90 °, acquisition matrix: 200 x 150, number of averages: 2, slice thickness: 0.7 mm, duration: 2 min 53 s). Further functional MR imaging included a *T*_2_* relaxometry using a multi-gradient echo sequence (MGE parameters: 3D sequence, 0.1 mm in-plane resolution, TE: 2.57 ms, TR: 73.43 ms, flip angle: 20°, acquisition matrix: 200 x 200, number of averages: 2, slice thickness: 0.1 mm, duration: 22 min 7 s). *T*_2_* relaxometry was used for T cell tracking using the iron oxide NP Biopal. For MRI, animals were anesthetized with 3% isoflurane. Anesthesia was maintained with 1 - 1.5% isoflurane. Animals were kept on a heating pad to maintain constant body temperature and respiration was monitored externally during imaging with a breathing surface pad controlled by a LabVIEW program developed in house (National Instruments Corporation).

### Analysis of MRI data

MR images were exported as DICOM files and visualized in using open-source Slicer Software (3D Slicer V. 4.10.2, www.slicer.org). Tumor volume was segmented semi-automatically using Slicer's Segment Editor module. *T*_2_* relaxation times were calculated from MGE raw data and exported as *T*_2_* relaxation maps in DICOM-format using a customized script (MATLAB R2020a, 64-bit version for Windows, MathWorks). Briefly, reconstruction of T2* relaxation maps included noise filtering with removal of all voxels exceeding four standard deviations of noise level of each dataset before fitting the data Mean T2*-relaxation time of the entire tumor volume was extracted for further analyses. Subsequent MRI time points were co-registered using Slicer's Elastix module.

### Tumor response criteria

Tumor response criteria were based on weekly MR imaging. For tumor monitoring, MRI imaging was performed 2 weeks after tumor inoculation (baseline, day 13), followed by tumor monitoring on day 3, 7, 10 and 14 after intracranial T cell injection. Classification of tumor response was assessed as the following: CR was defined as relative increase in lesion volume pre MRI - day 14 (%V pre MRI - MRI day 14) of -100%, PR as %V pre MRI - MRI day 14 ≤ -65.0%, SD as %V pre MRI - MRI day 14 > -65% and < +40%, and PD as (%V pre MRI - MRI day 14 ≥ +40%) 14.

### Isolation and NP labeling of T cells

After euthanizing donor mice, spleens were excised and meshed through a 70 μm cell strainer to obtain single-cell suspensions. Red blood cells were lysed with ACK buffer (150 mM NH_4_Cl, 10 mM KHCO_3_ and 100 μM Na_2_EDTA). CD8^+^ T cells were enriched using a mouse CD8a+ T Cell Isolation Kit (Miltenyi) and LS columns. Enrichment was performed with magnetic activated cell sorting (MACS) buffer consisting of 0.5% bovine serum albumin (BSA) and 2 mM Ethylenediaminetetraacetic acid (EDTA) in PBS. T cells were cultured in T cell proliferation medium: 10% FBS (Sigma Aldrich, S0615), 1% penicillin / streptavidine (Sigma Aldrich, P4333), 50 µM β-mercaptoethanol (Sigma Aldrich, M3148), 2 mM L-glutamine (Sigma Aldrich, 59202C), 25 mM HEPES (Sigma Aldrich, H0887), 1 mM sodium pyruvate solution (Gibco, 11360-070) and 0.1 mM non-essential amino acids (Sigma Aldrich, M7145) in RPMI 1640 (PAN-Biotech, P0418500) and stimulated with 20 U / mL IL-2, 1 µl / mL CD3 (Biozym; B102102) and 2 µL / mL CD28 (ThermoFisher; 14-0031-82) for 48 h. After 24 h, T cells were labeled with 25 µL / mL Molday ION^TM^ EverGreen (NP size: 35 nm, zeta potential: +31 mV; BioPhysics Assay Laboratory (BioPAL)) for 24 h. T cells were washed with PBS prior to adoptive cell transfer (ACT) to get rid of excess NP. 3x10^6^ CD8^+^ T cells were diluted in 3 µL sterile PBS (Capricorn, PBS-1A) and stereotactically injected intratumorally with 10 µL Hamilton micro-syringe driven by a fine step stereotactic device (Stoelting) using the coordinates from the initial tumor cell inoculation. For intraventricular injections, cells were injected 0.5 mm left from the Bregma and 1.8 mm deep. Intravenous injections were performed by slow T cell injection diluted in 100 µL PBS in the tail vein using a 30 g needle.

### Isolation of tumor-infiltrating lymphocytes

Mice were intracardially perfused after receiving a lethal dose of ketamine / xylazine. For Gl261 tumors, the cerebellum was removed and the tumor-bearing hemisphere was separated from the non-tumor bearing hemisphere and processed separately. After enzymatical digestion (HBSS, Sigma (Aldrich) with 50 µg / mL Liberase D (Roche)) for 30 min at 37 °C, the tissue was meshed twice through a 100 μm and 70 µm cell strainer to obtain single cell suspensions. Myelin removal was performed with a 30% Percoll gradient (GE Healthcare, Princeton, NJ, USA) according to the manufacturer's instruction.

### CAR T cell manufacturing

Cryopreserved human peripheral blood mononuclear cells from three healthy donors were thawed and activated with anti-CD3 (clone OKT3) and anti-CD28 (clone 28.2) antibodies (Biolegend) and cultured with IL-7 (10 ng / mL) and IL-15 (5 ng / mL) (R&D System). PBMCS were transduced on day three with the RVSFG.CD19.CD28.4-1BBzeta retroviral vector (kindly provided by M Brenner, Baylor College of Medicine, Houston, Texas) using spinoculation on retronectin-coated (3.5 µg / mL, Takara) 24-well plates. CAR-T cells were maintained in cytokine-supplemented media (ImmunoCult™-XF T Cell Expansion Medium, Stemcell Technologies) and used for experiments 10 days after transduction.

### Flow cytometry

For flow cytometry, cell suspensions were blocked with anti-CD16/CD32 (eBioscience; 93; 14-0161) and the fixable viability dye FVD e780. Extracellular targets were stained for 30 min at 4 °C using the antibodies listed in (**Table S1**). For intracellular cytokine staining, cells were incubated with ionomycin, PMA and 5 µg / mL Brefeldin A (Sigma-Aldrich) for 5 h at 37 °C, 5% CO_2_ to allow for intracellular enrichment of cytokines. Intracellular antigens were fixed, permeabilized and stained using the staining buffer set (eBioscience; 00-5523). Staining of intracellular targets was performed for 45 min at 4 °C. Stained cells were analyzed on FACS Aria II. LSR Fortessa (BD Biosciences; Germany) or on FACS Canto II. FlowJo V10.8.1 was used for data analysis.

### Isolation and differentiation of bone marrow-derived macrophages

Bone marrow-derived macrophages (BMDMs) were isolated as described previously 39. Briefly, bone marrow was flushed from tibiae and femurs of C57BL/6 wild-type (WT) adult female mice using ice-cold Hanks' balanced salt solution (HBSS) and filtered through a 70 μm cell strainer. For differentiation, cells were seeded in 6-well plates at a density of 350 000 cells/ml and grown in RPMI 1640-Glutamax medium (Life Technologies) supplemented with 10% of heat-inactivated fetal bovine serum (Hyclone; Thermo Scientific), 1% penicillin/streptomycin (Sigma-Aldrich), and 10 ng / mL M-CSF, macrophage colony-stimulating factor 1 (Sigma-Aldrich). On day 4 after isolation, nonadherent cells were discarded by washing with prewarmed PBS, and media was replaced daily for adherent BMDMs.

### Immunohistochemistry

For histological correlation analysis, mice were sacrificed in deep anesthesia by intracardial perfusion with PBS. Brains and spleens were snap frozen in Tissue-Tek® O.C.T.TM (Sakura). 7 µm thick sections were cut at a CM1950 cryotome (Leica). CD3 / CD90.1 co-staining was performed by fixing cryoslides with ice cold acetone (-20 °C) for 10 min. After washing slides were blocked with 0.1% tween and 4% normal goat serum in PBS for 1 h at room temperature (RT). Slides were incubated with primary anti-CD3^+^ antibody (Dako, 1 : 200 dilution in PBS with 0.1% tween and 4% normal goat serum) overnight, washed using 0.1% tween in PBS, incubated with Alexa-546nm secondary antibody (Thermo Fisher, 1 : 200 dilution in 0.1% tween and 4% normal goat serum) for 1 h, washed, incubated with anti-CD90.1+ (Thermo Fisher, 1 : 100 dilution in PBS with 0.1% tween and 4% normal goat serum) for 1 h and mounted with antifade mounting medium with DAPI (VECTASHIELD). Tile scans (10x of the entire tumor-bearing hemisphere) and higher-magnification images (40x) were acquired by confocal microscopy (Zeiss LSM700).

### Prussian blue staining

Cryoslides were thawed, fixed with ice cold methanol for 10 min and rinsed with PBS. Staining was performed using the Prussian Blue staining reagent pack by BioPAL (CL-01-50): Reagent A and B were mixed in equal parts for the working solution. Working solution was applied for 10 - 20 min. Slides were then rinsed with distilled water three times and counterstained using a 1 : 50 pararosaniline solution in H_2_O for 3 - 5 min. Lastly, slides were incubated in an ascending alcohol row of 50%, 70%, 96% and 100% EtOH, shortly incubated in xylol and mounted with Eukit. Images were acquired using the Axioscan microscope with a 20x objective.

### RTCA

5.000 Gl261-Ova tumor cells/well were seeded in RTCA e-plates (X) for in DMEM supplemented with 10 % fetal bovine serum (FBS) and 100 U / mL penicillin and 100 µg / mL streptomycin (all Sigma-Aldrich) at 37 °C, 5% CO_2_. After 24 h, 5.000, 10.000 or 100.000 CD8^+^ T cells were added per well. Cells were cocultured for 7 days. Tumor cell killing was analyzed with the RTCA software.

### LDH release assay

LDH release assay was performed using the assay by Cell Signaling. Gl261-Ova cells and OT-1 T cells were co-cultured in T cell proliferation medium in a 96 well U bottom plate overnight at 37 °C, 5% CO_2_. 10 µL of lysis solution was added to wells containing target cell maximum release control and volume correction and incubated for 45 min at 37 °C, 5% CO_2_. 50 µL samples were transferred to a 96 well flat bottom plate, 50 µL substrate was added and incubated for 30 min at RT. 50 µL stop solution was added and absorbance was measured at 490 nm.

### Inductively coupled plasma optical emission spectroscopy (ICP-OES)

Primary murine T cells were incubated with the NP (5 µg, 25 µg, 50 µg or 100 µg / 1x10^6^ cells) in TCPM for 24 h, centrifuged and washed with PBS. 1 mL of 65% nitric acid and 200 µL hydrogen peroxide were added to each cell sample to ensure cell lysis. Samples were heated at 90 °C for 1 h and 8.2 mL MilliQH_2_O was added for a total volume of 10 mL per sample. Analysis was done on Agilent ICP-OES 720.

### Tissue Clearing and light sheet microscopy (LSM)

Animals were perfused with 4% PFA and isolated brains were post-fixed with 4% PFA for 24 h and then stored at 4 °C until further processing. Fixed samples were processed according to the iDISCO protocol 40. All following steps were performed on a shaker. First, the samples were dehydrated with a methanol / PBS series for 1 h each at room temperature (RT): 40%, 60%, 80%, 100%, 100%. Followed by an overnight incubation in 66% Dichlormethan (DCM) (Carl Roth, KK47.1) and 33% methanol. The next day the samples were washed twice with 100% methanol and then bleached overnight in freshly prepared 5% H_2_0_2_ in methanol at 4 °C. In the next step the samples were rehydrated with methanol / PBS series and washed with PBS / 0.2% TritonX (PTx) for 1 h each at RT: 80%, 60%, 40%, 20%, PBS, PTx, PTx. For the Immunolabeling the samples were incubated in permeabilization solution for 2 d at 37 °C. Next the samples were blocked in blocking solution for 2 days. T-cells were labeled by CD3 (Dako, A0452). Samples were incubated with the primary antibody in PBS / 0.2%Tween 20 / 5% DMSO / 3% goat serum at 37 °C degrees for one week. Followed by four washing steps with PBS / 0.2%Tween 20 (PTw) until the next d and then incubated with the secondary antibodies in PBS / 0.2%Tween 20 / 3% Goat serum at 37 °C for one week and then washed in PTw four times until the next day. The samples were dehydrated with methanol / PBS series for 1 h each at RT: 40%, 60%, 80%, 100%, 100% and then incubated with 66% DCM / 33% methanol at RT. Followed by two washing steps in 100% DCM for 15 min. In the last step the samples were incubated, without shaking, in BABB (mixture of benzyl alcohol and benzyl benzoate in 1:2, Sigma-Aldrich, 24122-1L-M, W213802-1KG-K). Images were acquired with a light sheet microscope (LCS SPIM, Luxendo-Bruker; Heidelberg, Germany) with a 4x detection objective (Olympus XLFLUOR 4x / 340, 0.28 NA). Z-stacks were acquired using 5 µm step size at an effective magnification of 4.4x with pixel size of 1.48 x 1.48 µm. The following filter sets were used: laser 488 (emission filter wavelength between 497 - 554 nm), laser 561 (emission filter wavelength between 580 - 627 nm), laser 642 (emission filter wavelength between 655 - 704 nm), laser 685 (emission filter wavelength between 710 - 749 nm).

### Electron microscopy

Sample preparation was performed as recently described 41.: In brief, primary murine T cells were incubated with the iron oxide NP for 24 h *in vitro*, T cells were pelleted by 500 g 5 min centrifugation and fixed with 2.5% EM-grade glutaraldehyde and 2% EM-grade paraformaldehyde in 0.1 M Na-cacodylate buffer (pH 7.4) at RT for 5 min. Fixative was renewed and samples were incubated in at 4 °C overnight. T cells were rinsed with 0.1 M cacodylate buffer and incubated with 1% osmium tetroxide in dH_2_O for 20 min. Cells were washed in dH_2_O for 1 min four times and stained with 1% uranyl acetate in dH_2_O for 14 min. Afterwards, cells were rinsed in dH_2_O four times for 1 min each. Dehydration with an acetone series (50%, 70%, 90%, 2 x 100%) was performed for 45 s per step in the microwave. Cells were infiltrated with EPON epoxy resin 812 (hard formula) using increasing resin concentrations in 100% acetone (2 x 30%, 50%, 70%, 90%, 3 x 100% EPON in acetone). All infiltration steps were performed in the microwave for 3 min each at 24 °C. Cell pellets in 100% resin were transferred into an embedding mould, incubated at room temperature overnight, and subsequently polymerized at 60 °C for 2 days. Sections were made at 70 nm on a Leica UC7 ultramicrotome and imaged on a JEOL JEM-1400 flash electron microscope at 120 kV.

### Statistical analysis

Statistical analysis was performed with GraphPad Prism (version 9.3.1 for Windows, GraphPad Software, La Jolla, CA, USA). Significance was assessed by either unpaired t-test analysis when comparing two groups or one-way analysis of variance (ANOVA) analysis when comparing multiple groups. Data is shown as mean +/- SEM.

## Supplementary Material

Supplementary figures and table.
