# Supplementary material for: In vivo nanoparticle-based T cell imaging can predict therapy response towards adoptive T cell therapy in experimental glioma
Source: Theranostics. 2023 Sep 25;13(15):5170–82. doi: 10.7150/thno.87248 (PMC10614679; doi:10.7150/thno.87248)
Supplement: Supplementary file 1 — Supplementary figures and table. [file thnov13p5170s1.pdf]

- Suppl. information -

In vivo nanoparticle-based T cell imaging can predict therapy response towards adoptive T cell therapy in experimental glioma

Jessica Hunger[1,2], Katharina Schregel[1], Berin Boztepe[1,2], Dennis Alexander Agardy[2,3], Verena Turco[1,2,3], Kianush Karimian-Jazi[1], Ina Weidenfeld[1], Yannik Streibel[1], Manuel Fischer[1], Volker Sturm[1], Rachel Santarella-Mellwig[6], Michael Kilian[2,3], Kristine Jähne[2,3], Katharina Sahm[2,3], Wolfgang Wick[4,5], Lukas Bunse[2,3], Sabine Heiland[1], Theresa Bunse[2,3], Martin Bendszus[1], Michael Platten[2,3,7] and Michael O. Breckwoldt[1,2#]

[1]Neuroradiology Department, University Hospital Heidelberg, 69120 Heidelberg, Germany

[2]Clinical Cooperation Unit Neuroimmunology and Brain Tumor Immunology, German Cancer Consortium (DKTK) within the German Cancer Research Center (DKFZ), 69120 Heidelberg, Germany

[3]Department of Neurology, Medical Faculty Mannheim, Mannheim Center for Translational Neurosciences, Heidelberg University, Mannheim, Germany

[4]Clinical Cooperation Unit Neurooncology, DKTK within DKFZ, Heidelberg, Germany

[5]Department of Neurology, National Center for Tumor Diseases (NCT), Heidelberg University Hospital, Heidelberg, Germany

[6]European Molecular Biology Laboratory (EMBL), Heidelberg, 69117, Germany

[7]DKFZ-Hector Cancer Institute at the University Medicine Mannheim, Mannheim, 68167, Germany

[#] **Corresponding authors:**

Michael Breckwoldt, MD, PhD, Tel +49 6221 5636436, michael.breckwoldt@med.uni-heidelberg.de

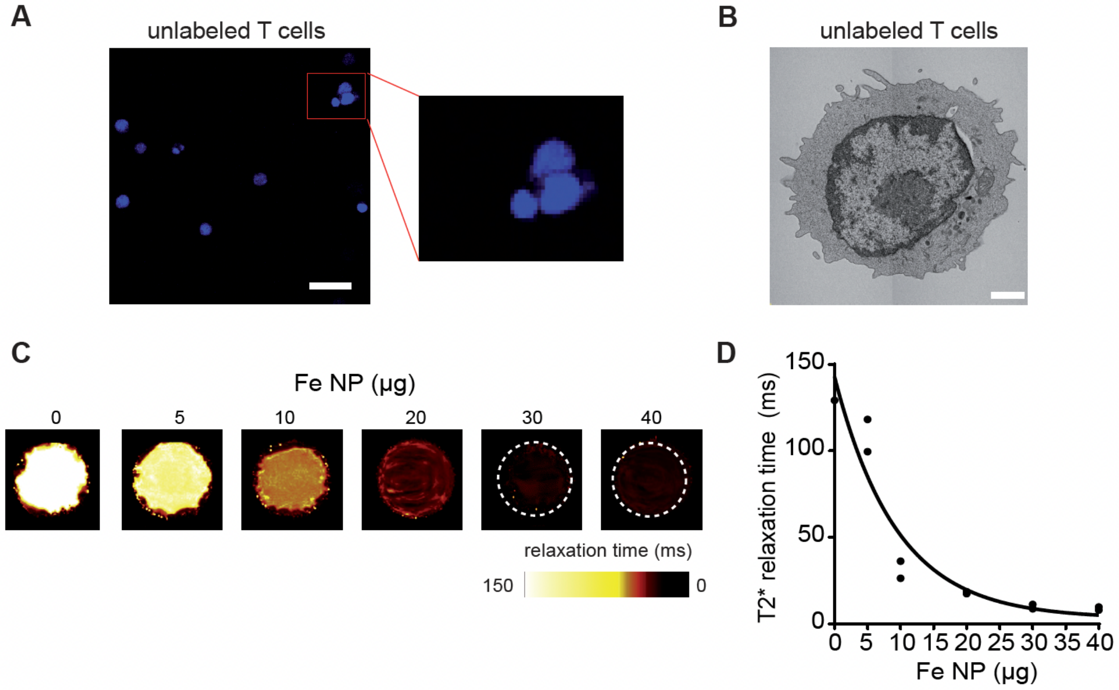

Figure S1

A: Confocal image of unloaded T cells as negative control **B:** TEM image of unlabeled primary murine T cell. **C:** T2* maps of 0 – 40 µg Fe of iron oxide NP **D:** Quantification of T_2* maps of 0 – 40 µg Fe of iron oxide NP. Scale bars are 20 µm in confocal microscopy image and 500 nm in EM.

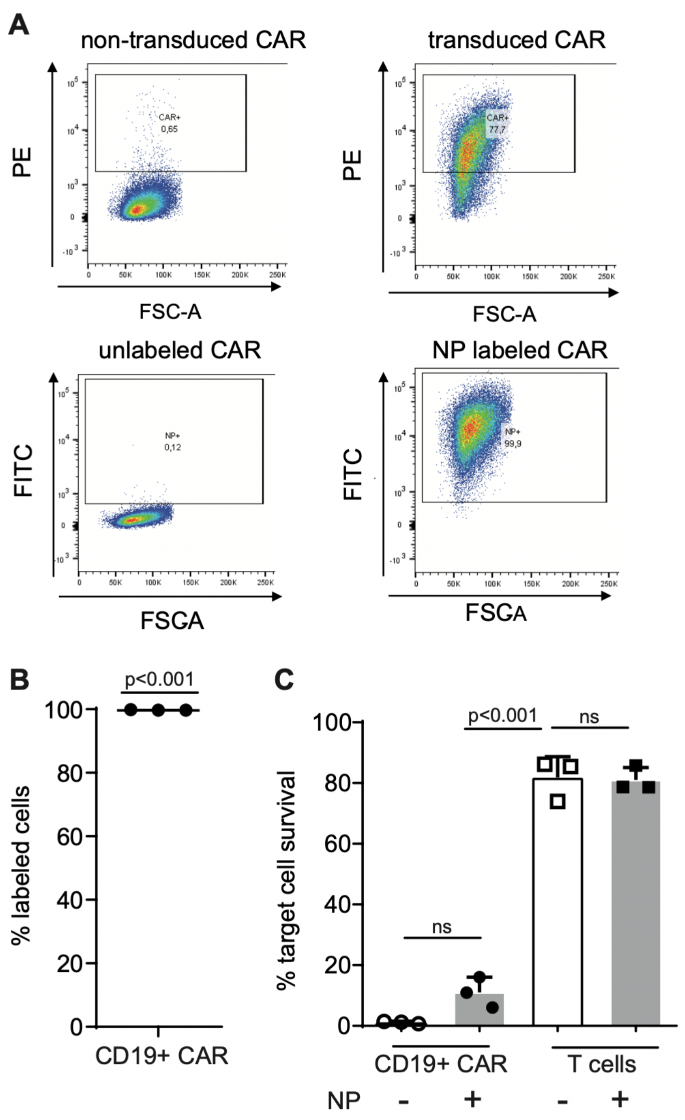

Figure S2

A: NP labeling of human CD19 CAR T cells and flow cytometry plots of labeling efficiency **B:** Quantification of labeling efficiency of human CD19 CAR T cells with iron oxide NP after 24 h as assessed by flow cytometry **C:** killing activity of unlabeled *vs* NP labeled human CD19 CAR T cells of NALM6 target cells in comparison to human T cells as assessed by flow cytometry.

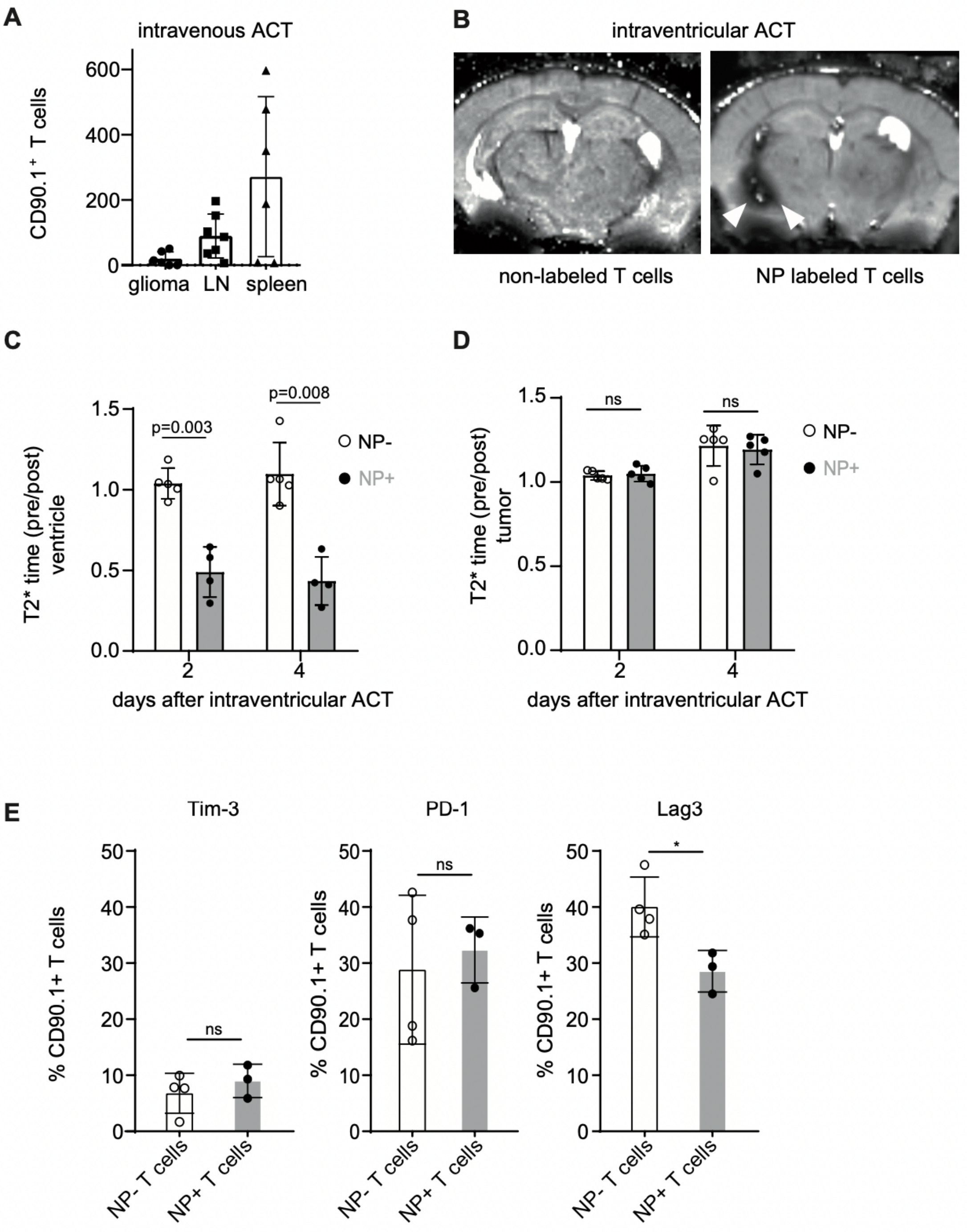

Figure S3

A: PMEL T cell infiltration after i.v. ACT in brain, lymph node and spleen as quantified by flow cytometry **B**: T2* maps of intraventricular ACT comparing unlabeled and iron oxide NP labeled T cell injection **C**: quantification of T2* maps of ventricles after intraventricular ACT after 2 and 4 days comparing unlabeled and iron oxide NP labeled T cells **D**: quantification of T2* maps of tumor area after intraventricular ACT after 2 and 4 d **E:** Tim-3, PD-1 and Lag-3 expression on adoptively transferred, CD90.1^{+} T cells after intratumoral ACT.

Table S1

Flow cytometry antibodies:

**Antibody**	**Company**	**Order number**
fixable viability stain eFluor 780	BD Horizon	565388
CD3 murine BV510	BioLegend	100233
CD8 AF700	BioLegend	100730
CD45.1 PE-Cy7	BioLegend	110730
CD45.2 APC	BioLegend	109814
CD90.1 PE-Cy7	BioLegend	202517
CD11b BV711	BioLegend	101241
PD-1 PerCP-eFl710	ThermoFisher	46-9985-82
Lag3 BV785	BioLegend	125219
Tim3 BV421	BioLegend	119723
CD3 human PE	BioLegend	300308
CD19 BV510	BioLegend	363020
R-Phycoerythrin AffiniPure $F(ab')_2$ Fragment Goat Anti-Human IgG (H+L)	Jackson ImmunoResearch	109-116-088
anti-CD16/CD32	eBioscience	93 14-0161
Counting beads	Invitrogen	01-1234-42

Antibodies used for clearing:

**Antibody**	**Company**	**Order number**
CD3 (rabbit anti-mouse/human)	Dako	A0452
goat anti-rabbit Alexa 568	Thermofisher	A11011
